# Yangyin Yiqi Mixture Ameliorates Bleomycin-Induced Pulmonary Fibrosis in Rats through Inhibiting TGF-β1/Smad Pathway and Epithelial to Mesenchymal Transition

**DOI:** 10.1155/2019/2710509

**Published:** 2019-01-03

**Authors:** Lihong Meng, Xiaomei Zhang, Hong Wang, Huan Dong, Xiaofeng Gu, Xiaolin Yu, Yushan Liu

**Affiliations:** ^1^Beijing University of Chinese Medicine, No. 11 on North 3rd Ring Road, Beijing 100029, China; ^2^Department of Respiratory Medicine, Dongfang Hospital Affiliated to Beijing University of Chinese Medicine, No. 6 on 1st District of Fangxingyuan, Beijing 100078, China; ^3^Beijing Chinese Medicine Hospital, Shunyi Hospital, No. 5, Zhanqian East Street, Shunyi District, Beijing 101300, China; ^4^Beijing Chinese Medicine Hospital Affiliated to Capital Medical University, No. 23, Houjie Street, Art Museum, Dongcheng District, Beijing 100010, China

## Abstract

**Objective:**

The aim of the current study was to investigate the protective effect of Yangyin Yiqi Mixture (YYYQ) on Bleomycin-induced pulmonary fibrosis in rats based on TGF-*β*1/Smad signal pathway and epithelial to mesenchymal transition (EMT).

**Methods:**

120 Wistar rats were randomly divided into six groups: control group, BLM group, BLM + Pred group, BLM+YYYQ-L group, BLM+YYYQ-M group, and BLM+YYYQ-H group. Rats were given an intratracheal instillation of 3 mg/kg BLM to establish the pulmonary fibrosis model and followed by different dosages of YYYQ (11, 22, 44g/kg, via intragastric gavage) or prednisone soluble (4.2mg/kg, via intragastric gavage) or water. After 14 days and 28 days, tissue sections were stained with hematoxylin-eosin and Masson's trichrome to observe histopathological changes. Protein levels of TGF-*β*1, CTGF, Interleukin 18, and hydroxyproline were detected by ELISA method, and mRNA expressions of TGF-*β*1, T*β*RI, T*β*RII, Smad3, Smad7, *α*-SMA, E-cadherin, laminin, and collagen I were detected by RT-PCR.

**Results:**

TGF-*β*1, CTGF, Interleukin 18, and hydroxyproline levels and mRNA expression of TGF-*β*1, T*β*RI, T*β*RII, Smad3, *α*-SMA, laminin, and collagen I were significantly increased (*p* <0.01), while Smad7 and E-cadherin levels were significantly decreased in BLM group (*p* <0.01). YYYQ-M and YYYQ-H group had downregulated the TGF-*β*1, CTGF, hydroxyproline contents, and mRNA expression of TGF-*β*1, T*β*RI, T*β*RII, Smad3, *α*-SMA, laminin, and collagen I and upregulated mRNA levels of Smad7 and E-cadherin significantly (*p* <0.01 or* p* <0.05). The result from the present study, which was also supported by histological evidence, suggested that YYYQ-M group and YYYQ-H group exhibited better treatment effect on Bleomycin-induced pulmonary fibrotic rats when compared to that of BLM + Pred group (*p* <0.01). Meanwhile, the effect of YYYQ, in three different dosages, on the level of interleukin 18 was not significant.

**Conclusion:**

These results showed that YYYQ has the potential of ameliorating the progression of pulmonary fibrosis, and the mechanism may be related to suppressing TGF-*β*1/Smad signal pathway and EMT in BLM-induced pulmonary fibrosis of rats.

## 1. Introduction

Idiopathic pulmonary fibrosis (IPF) is a life-threatening disease, which is characterized by chronic inflammatory response, excessive proliferation of fibroblasts, aberrant deposition of extracellular matrix (EMC), and abnormal repair and remodeling of lung tissue [1]. Studies showed that many causes, such as environmental pollution, bacterial infections, smoking, and gastroesophageal reflux, are closely related to the onset of IPF [2–5]. Since IPF can deteriorate lung functions, which may then lead to respiratory failure and eventually death, the prognosis of IPF is usually poor. Although studies had reported that combination of glucocorticosteroids (such as prednisone), azathioprine, and N-acetylcysteine was unable to decrease the mortality and morbidity rate of IPF, such combination remained as the conventional treatment for IPF patients in order to relieve inflammation, suppress immune responses, and relieve signs and symptoms during the course of the disease [6]. Pirfenidone and nintedanib are the two newly developed antipulmonary fibrotic drugs which can significantly improve lung function and delay the progression of IPF. However, the usage of such drugs is often limited in clinical settings due to its high price and side effects [7, 8]. Hence, it is necessary that further research is to be carried out to explore new treatment methods for IPF.

Transforming growth factor-*β*1 (TGF-*β*1) is a well-recognized profibrotic factor, which plays a crucial role in resulting multiple organs fibrosis through increasing the production of glucose and proteoglycans, promoting the transport of amino acids, and promoting the deposition of extracellular matrix [9], among which, Smad-mediated TGF-*β*1 is regarded as one of the most important signaling pathway in causing fibrosis. Meanwhile collagen (type I and type II), fibronectin, hydroxyproline, and laminin are the few main components of extracellular matrix [10, 11]. Lucarini L. et al. [12] found that inhibition of TGF-*β*/Smad pathway could significantly improve the static compliance of airway and lung, reduce lung tissue hardness, and downregulate the expression levels of inflammatory factors such as tumor necrosis factor-*α*, interleukin 1*β*, iNOS, and COX-2, reduce *α*-SMA expression in BLM-induced pulmonary fibrosis in mice, and ameliorate the progression of signs and symptoms of pulmonary fibrosis.

Fibroblasts and myofibroblasts, which can be resulted via activation of original fibroblasts, proliferation, and differentiation of bone marrow-derived stem cells or epithelial-mesenchymal transition (EMT), are the main effector cells that produce extracellular matrix in pulmonary fibrosis [13]. This is in agreement with Tanjore et al. [14], in which the team reported that about one-third of fibroblasts in lung tissues of BLM-induced pulmonary fibrotic mice was derived from type II lung epithelial cells through the process of EMT. EMT is characterized by low expression of epithelial cell markers such as E-cadherin and ZO-1 and overexpression of mesenchymal markers such as *α*-Smooth muscle actin (*α*-SMA) and vimentin [15, 16]. Meanwhile, TGF-*β*1 is regarded as an important triggering factor for the onset of pulmonary fibrosis by accelerating the process of transformation of alveolar epithelial cells into mesenchymal myofibroblasts [17, 18]. The study conducted by Ji Y. et al. further supported this statement as a result of their study proved that TGF-*β*1 stimulated the morphogenesis of A549 cells and induced EMT [19]. In a nutshell, the levels of SMA, vimentin, and types I and III collagen can be decreased by inhibiting the TGF-*β*1/Smad pathway. On the other hand, E-cadherin expression can be further enhanced by inhibiting TGF-*β*1/Smad pathway.

From the perspective of traditional Chinese medicine, deficiency of qi and yin, and stasis of blood and phlegm formed the main pathogenesis of IPF [20]. YYYQ is an effective prescription created by Wen Zhenying, a national famous doctor of Chinese medicine. The main function of YYYQ is to replenish qi and nourish yin and promote blood circulation and detoxification, and the curative effect of YYYQ against IPF has been proven in both clinical and experimental studies [21, 22]. The aim of our study was to explore the protective effect of YYYQ on BLM-induced pulmonary fibrosis in rats based on TGF-*β*1/Smad signal pathway and EMT.

## 2. Materials and Methods

### 2.1. Animals

120 adult SPF Wistar rats (male, 6 weeks old, weighting around 200g) were purchased from Vital River Laboratory Animal Technology Co., Ltd., Beijing, China, and housed in laboratory animal center of Dongfang Hospital, Beijing University of Chinese Medicine. All rats were kept at a constant temperature of 25°C and humidity of 45%-55% with a 12-hour light-dark cycle and free access to diet and tap water. The experiment protocol was approved by the Ethical Committee for the Experimental Animals at Dongfang Hospital, Beijing University of Chinese Medicine.

### 2.2. Chemicals and Reagents

YYYQ consists of astragalus mongholicus (huangqi, 15g),* Codonopsis pilosula* (dangshen, 15g), radix scrophulariae (xuanshen, 15g), radix glehniae (beishashen, 15g), rhizome polygonati (huangjing, 10g), radix lithospermi (zicao, 10g), ligusticum wallichii (chuanxiong, 10g), smoked plum (wumei, 10g), radices trichosanthis (tianhuafeng, 10g), and pericarpium citri reticulatae (chenpi, 10g). All the herbs are measured up to the criteria of the Pharmacopoeia of the People's Republic of China (2015 Edition). The decoction was made by Preparation Center of Dongfang Hospital from crude herbs. All the herbs were soaked in water for 1 hour and the mixture was boiled for half an hour for extracted solution for the first time. Then, the herbs were decocted for 20 minutes again after immersing for 30 minutes. The decoction of this was mixed twice together and then further concentrated to 2 g/ml.

Bleomycin (BLM) was provided from Haisun Pfizer pharmaceuticals Co. Ltd., Zhejiang, China. Prednisone was obtained from Lisheng Pharmaceutical Co., Ltd., Tianjin, China. TRIzol reagent was purchased from Tian Gen biotechx Co., Ltd., Beijing, China. PrimeScript RT reagent kit with gDNA Eraser was purchased from Takara Biotechnology, Co., Ltd., Beijing, China. TGF-*β*1 kit was purchased from R&D Systems, Inc. Connective tissue growth factor (CTGF) and interleukin 18 kits were purchased from Abbexa. Hydroxyproline kit was obtained from Cell Biolabs.

### 2.3. Experiment Protocol and Model Establishment

Rats were anesthetized with abdominal injection of 1% pentobarbital sodium (45mg/kg) and given an intratracheal instillation of 3 mg/kg BLM, followed by immediate turning of the rats to ensure thorough drug distribution in the lungs. The rats were randomly divided into six different groups (n=20 per group), namely, BLM group, BLM+ Pred group, BLM+ YYYQ-L group, BLM+ YYYQ-M group, and BLM+ YYYQ-H group. The dosage of medication given to the rats was calculated according to the conversion of animal dose to human equivalent doses based on body surface area [23]. Rats in six different groups were intervened as follows: (1) control group and (2) BLM group: given the same volume of normal saline; (3) BLM+ Pred group: gavage with prednisone suspension 4.2mg/kg every day; (4) BLM+ YYYQ-L group (5) BLM+ YYYQ-M group and (6) BLM+ YYYQ-H group: gavage with YYYQ mixture at doses of 11 g/kg, 22 g/kg, and 44g/kg every day. On day 14 and day 28 after initial instillation, eight rats from each group were dissected randomly, with their lungs removed. The left lungs were fixed with 4% paraformaldehyde for histopathological examination. Meanwhile, the right lungs were stored in -80°C for RT-PCR assay and the sera were collected and stored in -20°C for ELISA assay.

### 2.4. Lung Histopathology Examination

The paraffin-embedded pulmonary specimens were sectioned into 4 *μ*m thick slices and then deparaffinized in xylene for 15 min, hydrated in gradient alcohol, and then rinsed with 1% phosphate-buffered saline three times. Then these slices were stained with hematoxylin and eosin (H&E) and Masson's trichrome for morphologic detection. The H&E staining was used to assess the alveolitis inflammation and Masson's staining was used to observed degree of lung fibrosis. Alveolitis and pulmonary interstitial fibrosis were quantified by Szapiel scoring system [24].

### 2.5. Enzyme-Linked Immunosorbent Assay (ELISA)

TGF-*β*1, CTGF, interleukin 18, and hydroxyproline kits were used to detect the protein expression of TGF-*β*1, CTGF, interleukin 18, and hydroxyproline in sera of rats according to the manufacturer's protocols.

### 2.6. Real-Time Polymerase Chain Reaction (RT-PCR)

Total RNA in the lung was extracted using the TRIzol reagent and reverse transcribed to cDNA using the Prime Scriptr RT reagent kit according to the manufacturer's instructions. RT-PCR was performed on the cDNA samples using the SYBRr Premix Ex TapTM II. The reaction conditions were as follows: 95°C for 30 s, followed by 40 cycles of denaturation at 95°C for 5 s and extension at 60°C for 34 s. All amplifications were done in triplicate and repeated three times. The data were analyzed by the QuantStudio 7 Flex detection system. Cycle threshold (CT) values were analyzed by the comparative CT (ΔΔCT) method, and the relative amount of target mRNA (2^−ΔΔ CT^) was obtained by normalizing to endogenous glyceraldehyde 3-phosphate dehydrogenase (GAPDH). Information of RT-PCR primers is shown in Table 1.

### 2.7. Statistical Analysis

All the data in the study were expressed as mean ± standard deviation (SD) and analyzed with SPSS 20.0. One-way analysis of variance (ANOVA) and least significant difference (LSD) test were used to analyze the data with normal distribution, and Tamhane's T2 test was used to evaluate the nonnormally distributed continuous data. Value* p* < 0.05 was considered as a significant difference in statistics.

## 3. Results

### 3.1. YYYQ Attenuated BLM-Induced Pulmonary Fibrosis in Rats

**Figure 1:**
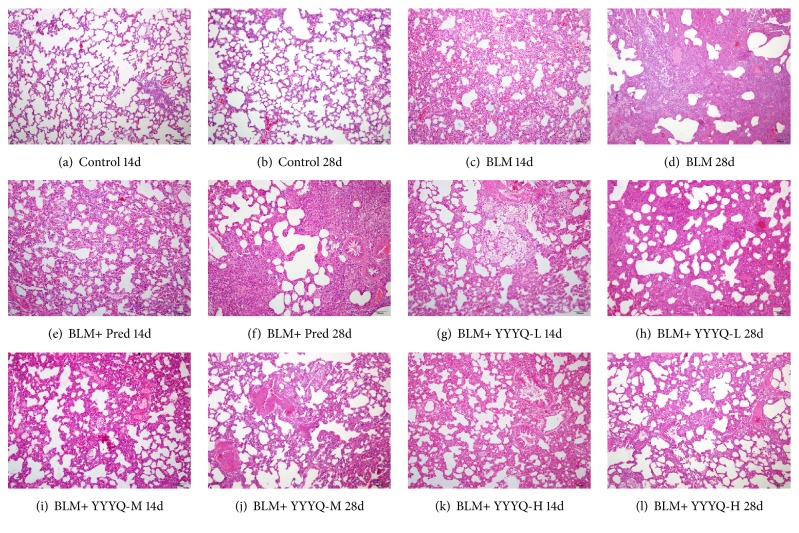
H&E staining of lung tissues (100x). (a) Control group treated by normal saline on days 14. (b) Control group treated by normal saline on days 28. (c) BLM group induced pulmonary fibrosis and treated by normal saline on days 14. (d) BLM group induced into pulmonary fibrosis and treated by normal saline on days 28. (e) BLM + Pred group treated by prednisone on days 14. (f) BLM + Pred group treated by prednisone on days 28. (g) BLM + YYYQ-L group treated by low dose of YYYQ on days 14. (h) BLM + YYYQ-L group treated by low dose of YYYQ on days 28. (i) BLM + YYYQ-M group treated by middle dose of YYYQ on days 14. (j) BLM + YYYQ-M group treated by middle dose of YYYQ on days 28. (k) BLM + YYYQ-H group treated by high dose of YYYQ on days 14. (l) BLM + YYYQ-H group treated by high dose of YYYQ on days 28.

H&E staining was used to observe the changes of alveolar inflammation in lung tissues. The results are shown in Figure 1. Under the microscope, samples in control group indicated that the alveolar wall was intact, the alveolar cavity was clear, no bleeding or exudate was found in the alveolar and bronchial cavity, and no obvious inflammatory cells infiltration was observed on day 14 and day 28. On day 14, samples from BLM group showed that alveolar cavity and part of bronchi structure had collapsed and destructed, alveolar septal became thick and swollen, and large amount of inflammatory cells infiltration was observed in pulmonary interstitial tissues and alveolar tissues. On day 28, pulmonary interstitial inflammation was reduced, while the alveolar structure was replaced by collagen fibers because of the fibroblast proliferation. In summary, the histopathological results showed that lesions of lung tissues from the rats in BLM group and all the treatment groups were more severe compared to that of rats in control group. However, thickness of alveolar septa and infiltration of inflammatory cells among the rats in BLM + Pred group and different dosages of YYYQ groups was significantly reduced when compared to that of rats in BLM group.

**Figure 2:**
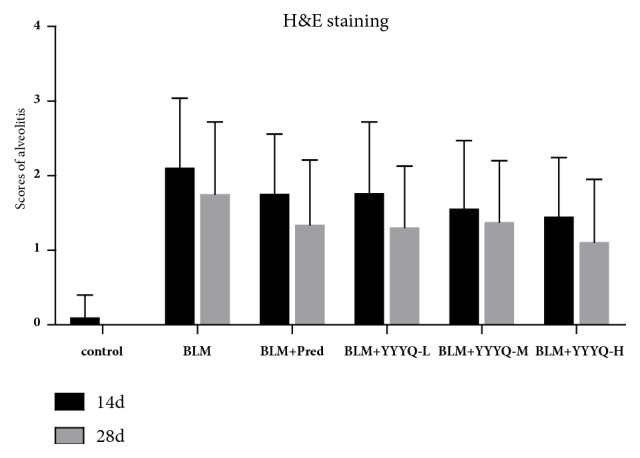
Scores of alveolitis.

H&E score is shown in Table 2 and Figure 2. On days 14 and 28, the alveolitis scores in all groups treated with BLM were higher than that in control group (*p* <0.01), whereas compared with BLM group, BLM+YYYQ-M group and BLM+YYYQ-H group have lower alveolitis scores (*p* <0.01). However, BLM + Pred group did not show an advantage in improving pulmonary fibrosis alveolitis than YYYQ groups.

**Figure 3:**
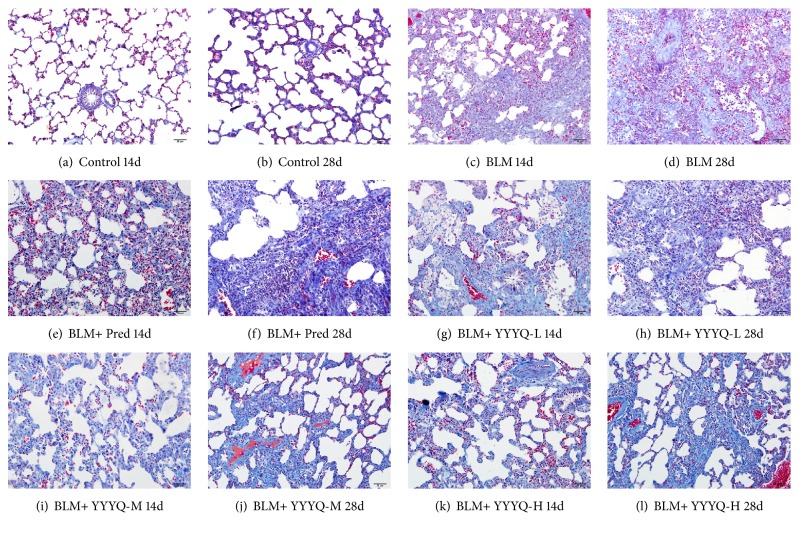
Masson's staining of lung tissues (200x). (a) Control group treated by normal saline on days 14. (b) Control group treated by normal saline on days 28. (c) BLM group induced pulmonary fibrosis and treated by normal saline on days 14. (d) BLM group induced into pulmonary fibrosis and treated by normal saline on days 28. (e) BLM + Pred group treated by prednisone on days 14. (f) BLM + Pred group treated by prednisone on days 28. (g) BLM + YYYQ-L group treated by low dose of YYYQ on days 14. (h) BLM + YYYQ-L group treated by low dose of YYYQ on days 28. (i) BLM + YYYQ-M group treated by middle dose of YYYQ on days 14. (j) BLM + YYYQ-M group treated by middle dose of YYYQ on days 28. (k) BLM + YYYQ-H group treated by high dose of YYYQ on days 14. (l) BLM + YYYQ-H group treated by high dose of YYYQ on days 28.

Masson's trichrome staining was used to observe the degree of pulmonary fibrosis, in which the microscopic images are illustrated in Figure 3. Control group showed that normal lung tissue structure and slight collagen deposition in the alveolar septa. On the other hand, BLM group indicated severe collagen deposition, obliteration of interalveolar septum, and damage of lung structure, whereby the alveolar space was replaced by collagen fibrosis and normal alveoli was further deteriorated on day 28. The results also indicated that the level of destruction of pulmonary interstitium and severity of collagen fibrosis in the alveolar septa were reduced in BLM + Pred group and different dosage of YYYQ groups.

**Figure 4:**
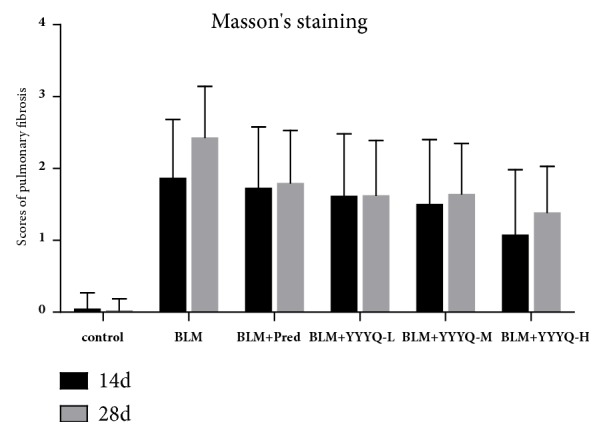
Scores of pulmonary fibrosis.

Masson's score is shown in Table 2 and Figure 4. The scores of pulmonary fibrosis in all groups treated with BLM were higher than control group on days 14 and 28 (*p* <0.01), and the event of interstitial fibrosis was more severe on day 28, indicating that pulmonary fibrosis was mainly observed on day 28. On day 14, BLM+YYYQ-H group significantly inhibited the progression of pulmonary fibrosis (*p* <0.01). On day 28, the fibrosis scores for all treatment groups were significantly lower than BLM group (*p* <0.01). Meanwhile, BLM+YYYQ-H group showed better treatment effect than BLM + Pred group (*p* < 0.05).

### 3.2. YYYQ Decreased the Protein Expression of TGF-*β*1, CTGF, Interleukin 18, and Hydroxyproline in Sera

**Figure 5:**
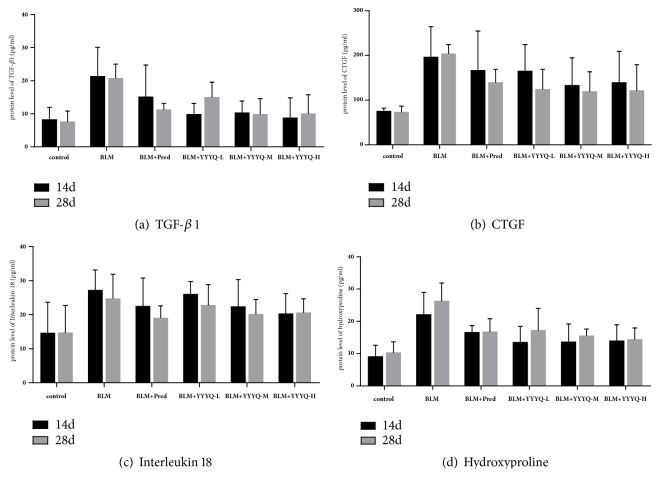
The secreted protein levels of TGF-*β*1, CTGF, interleukin 18, and hydroxyproline in each group of rats on days 14 and 28.

ELISA was used to detect the levels of protein concentration of TGF-*β*1, CTGF, interleukin 18, and hydroxyproline in the sera, and the results are shown in Table 3 and Figures 5(a)–5(d). On days 14 and 28, the levels of protein concentration of TGF-*β*1, CTGF, and hydroxyproline in BLM group were significantly higher when compared to that of control group (*p* < 0.01), whereas the levels of protein concentration of CTGF and hydroxyproline in BLM+ Pred group were significantly higher than that of control group (*p* <0.05). On day 28, the levels of protein concentration of TGF-*β*1 and hydroxyproline in YYYQ-L group were significantly higher than that in the control group (*p* <0.05) but were lower than that in BLM group (*p* <0.05). On day 28, the levels of protein concentration of TGF-*β*1, CTGF, and hydroxyproline in the sera samples of BLM+ Pred and BLM+YYYQ groups were significantly decreased compared to that of BLM group, especially in YYYQ-M and YYYQ-H groups (*p* < 0.01 or* p* < 0.05).

On days 14 and 28, the levels of protein concentration of interleukin 18 in BLM group and BLM+YYYQ-L group were significantly higher than that in control group (*p* < 0.01 or* p* < 0.05). The levels of protein concentration of interleukin 18 in other treatment groups were decreased when compared to that of BLM group, but were not statistically significant (p > 0.05).

### 3.3. YYYQ Inhibited BLM-Induced EMT in Rats

**Figure 6:**
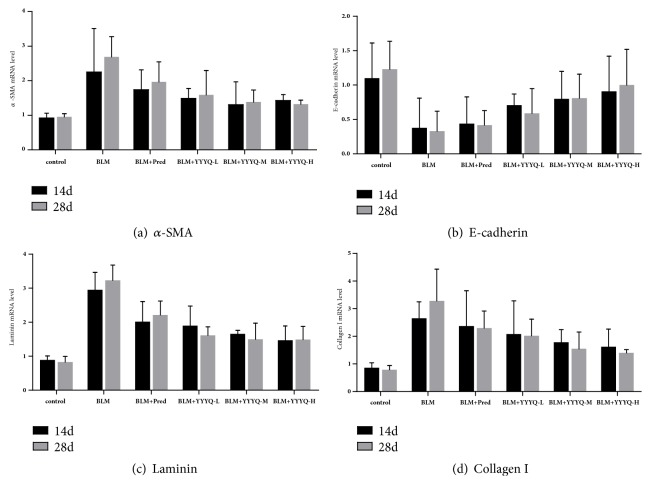
The mRNA expression of *α*-SMA, E-cadherin, laminin, and collagen I in each group of rats on days 14 and 28.

As shown in Table 4 and Figures 6(a)–6(d), the results indicated that, on day 14, compared with control group, the mRNA expression of *α*-SMA in BLM group was significantly higher (*p* < 0.01), while the mRNA expression of E-cadherin was significantly lower (*p* < 0.05). On day 28, the mRNA expression of *α*-SMA in BLM group and BLM + Pred group was significantly upregulated (*p* < 0.01), whereas mRNA expression of E-cadherin was significantly downregulated (*p* < 0.01). Treatment by prednisone and different dosage of YYYQ could reduce the mRNA expression of *α*-SMA compared with BLM only (*p* <0.05 or* p* <0.01). Meanwhile, mRNA expression level of E-cadherin in BLM+ YYYQ-H group was significantly upregulated compared to BLM group (*p* < 0.05), and it revealed more remarkable improvement than BLM+ Pred group (*p* < 0.05).

On both day 14 and day 28, mRNA expression of laminin and collagen I in BLM group, BLM + Pred group, and BLM + YYYQ-L group were significantly upregulated when compared to that of control group (*p* < 0.05 or* p* < 0.01). In summary, mRNA expression of laminin in all the treatment groups were significantly downregulated compared to BLM group on both day 14 and day 28 (*p* < 0.01); especially on day 28, mRNA level of laminin in different dosage of YYYQ group was significantly decreased compared with BLM + Pred group. The mRNA expression of collagen I in all the treatment groups was significantly downregulated when compared to that of BLM group (*p* < 0.05 or* p* < 0.01).

### 3.4. YYYQ Suppressed BLM-Activated TGF-*β*1/Smad Signaling Pathway in Rats

**Figure 7:**
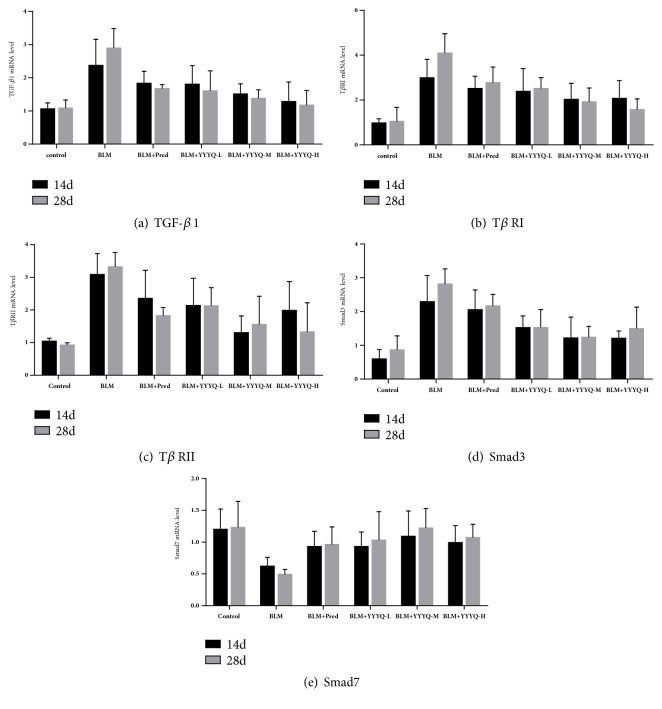
The mRNA expression of TGF-*β*1, T*β*RI, T*β*RII, Smad3, and Smad7 in each group of rats on days 14 and 28.

As indicated in Table 5 and Figures 7(a)–7(e), we observed that mRNA expression of TGF-*β*1, T*β*RI, and T*β*RII in BLM, BLM+ Pred, and BLM+YYYQ-L group were significantly higher than that of control group on both day 14 and day 28 (*p* <0.01 or* p* <0.05). On day 14, BLM+YYYQ-M and BLM+YYYQ-H group had downregulated mRNA expression of TGF-*β*1, T*β*RI, and T*β*RII (*p* <0.05). Meanwhile, mRNA expression of T*β*RII was significantly downregulated in YYYQ-M group than that of BLM + Pred group (*p* <0.05). On day 28, the mRNA expression of TGF-*β*1, T*β*RI, and T*β*RII in all treatment groups was significantly downregulated when compared to that in BLM group (*p* <0.01). The overall results indicated that the BLM+YYYQ-H group had significant advantage over the BLM+ Pred and BLM+YYYQ-L group (*p* <0.05).

On days 14 and 28, the mRNA expression of Smad3 in BLM and BLM+ Pred group was significantly upregulated (*p* <0.05 or* p* <0.01), whereas mRNA level of Smad7 was significantly downregulated in BLM group when compared to that of control group (*p* <0.01). BLM+YYYQ group could significantly downregulated the mRNA expression of Smad 3 than BLM group (*p* <0.05 or <0.01). Moreover, the mRNA level of Smad 3 in BLM + YYYQ-M group and BLM + YYYQ-H group was lower than BLM + Pred group (*p* < 0.05). Apart from that, the results also showed that mRNA expression of Smad 7 was significantly upregulated in all treatment groups, especially in BLM + YYYQ-M group (*p* < 0.05).

## 4. Discussion

IPF is a chronic, progressive, and irreversible lethal lung disease, and the annual incidence of IPF is rising. The main clinical manifestations of pulmonary fibrosis are cough, scanty phlegm, wheezing, fatigue, cyanosis, acropachy, etc. It can cause massive deterioration to the quality of life among the patients. So far, IPF still lacks safe and effective therapeutic regimens in the clinic. From the perspective of Chinese medicine, the main pathogenesis during the early stage of IPF, also known as Feibi, is invasion of exogenous pathogens into the lungs as a result of deficiency of lung qi, which then lead to formation of blood stasis in the lungs. If left untreated, the disease will gradually progress into late stage, also known as Feiwei, which is marked by deficiency of qi and yin. YYYQ has the effects of nourishing yin and benefiting qi, promoting blood circulation and removing toxic substances. Thus, the indications of YYYQ coincide with the main pathogenesis of IPF, which is marked by the presence of blood stasis and deficiency of qi and yin.

YYYQ contains astragalus mongholicus,* Codonopsis pilosula*, radix scrophulariae, radix glehniae, rhizome polygonati, radix lithospermi, ligusticum wallichii, smoked plum, radices trichosanthis, and pericarpium citri reticulatae. It is reported that astragalus, the main ingredient of YYYQ, has various main active ingredients, such as astragaloside, total saponin of astragalus, and astragalus polysaccharides, which have the potential to regulate cell apoptosis and cell differentiation by regulating TGF-*β*/Smad, Wnt/*β*-catenin, PI3K/Akt, and other signaling pathways in order to reduce oxidation, inflammation, and extracellular matrix deposition of the relative tissues [25]. Meanwhile, radix glehniae is reported to be able to enhance the phagocytosis of macrophages, increase the number of T lymphocytes, enhance the functions of immune system, and reduce the expression of collagens such as hydroxyproline, fibronectin, and laminin, which can be found in pulmonary fibrotic rats [26, 27]. It is also found that other ingredients of YYYQ, such as radix lithospermi, can inhibit the expression of endothelial growth factors to reduce angiogenesis, thereby preventing the progression of pulmonary fibrosis [28], whereas ligustrazine can decrease TGF-*β*1 and CTGF levels in pulmonary fibrotic rats [29].

In the present study, we used the classic method of intratracheal instillation of BLM to establish pulmonary fibrosis model of rats, the histopathologic changes, and molecular pathway of which are similar to human beings [30] and then intervention with the corresponding drugs occured. Then the protective effect of YYYQ on BLM-induced pulmonary fibrosis in rats by observing pathological changes and related protein and gene expression was investigated. The results of histopathological analysis showed that, during the early stage of pulmonary fibrosis, the main pathological manifestation of lung tissues was marked by alveolitis, which then developed into pulmonary fibrosis during the latter stage. On both day 14 and day 28, YYYQ has significantly reduced the degree of severity of alveolitis, which in turn delays the progression of alveolitis into pulmonary fibrosis, in which BLM + YYYQ-H group indicated better result compared to that of BLM + Pred group.

TGF-*β*1 played a key role in the progression of pulmonary fibrosis by promoting proliferation and differentiation of fibroblasts, enhancing synthesis of collagen, and altering some components in EMC [31]. Smad proteins are the main signal transducers for TGF-*β*1 signaling pathway [32], in which Smad3 binds to Smad 4 to form a complex and then make an entry into the nucleus in order to regulate the targeted gene transcription, which thereby interfere with the formation of lung fibrosis [33]. On the contrary, Smad7 inhibits pulmonary fibrosis by competitively binding to TGF-*β*1 and its receptors complex with Smad3 [34].On both day 14 and day 28, it is discovered that the mRNA expression of TGF-*β*1 and its receptors and Smad3 was significantly downregulated, while the mRNA expression of Smad7 was significantly upregulated. These results could hint the activation of TGF-*β*1/Smad signaling pathway during the process of pulmonary fibrosis. Although both YYYQ and prednisone resulted in downregulation of mRNA expression of TGF-*β*1 and its receptors and Smad3, as well as upregulation of Smad7, the results from the present studies indicated that BLM + YYYQ groups had a more significant treatment effect compared to that of BLM+ Pred group, which was consistent with histopathological results. The results above suggested that YYYQ could ameliorate the progression of PF by inhibiting TGF-*β*1/Smad pathway in the lungs of pulmonary fibrotic rats.

TGF-*β*1 is an important profibrotic factor, and CTGF is a direct downstream mediator of TGF-*β*1. During the process of pulmonary fibrosis, the increase of TGF-*β*1 induces the upregulation of CTGF, which promotes the proliferation and differentiation of fibroblasts and collagen production [35]. Although the role of interleukin 18 in the development of pulmonary fibrosis is yet to be conclusive, however, it is reported that interleukin 18 may act as a proinflammatory factor by secreting interleukin 1*β*, tumor necrosis factor-*α*, and other cytokines, which can aggravate the damage to the lung tissues. Apart from that, interleukin 18 can also promote the secretion of cytokines, such as interferon-*γ* and interleukin 2 by Th1 cells, which then enhances type 1 hypersensitive responses to delay the further progression of pulmonary fibrosis [36, 37]. The level of a specific amino acid found in collagen, known as hydroxyproline, could reflect the level of collagen fiber and ECM. The result of this study showed that YYYQ not only lower the protein concentration of TGF-*β*1 and CTGF, but also can lower the protein concentration of hydroxyproline in the sera of BLM-stimulated rats. The data also reflected that YYYQ lowered the protein concentration of interleukin 18, but the difference was not statistically significant.

Studies found that TGF-*β*1 can induce the process of EMT, which serve as an important pathogenesis in the development of pulmonary fibrosis [38–40]. Therefore, it can also be said that EMT can be suppressed by inhibiting the TGF-*β*1/Smad signaling pathway in order to delay the progression of fibrosis-related diseases [41]. E-cadherin, as an epithelial phenotype marker, has the function of maintaining epithelial constitutive and polar protein. Meanwhile, *α*-SMA and Collagen I are interstitial marker protein, which are secreted by myofibroblasts [42, 43], and they are main indicators of the content of extracellular matrix. During the process of EMT, the E-cadherin level is downregulated and the expression of *α*-SMA and Collagen I are upregulated [44, 45]. Laminin has the effects of inducing, adhering, stimulating growth, and differentiation on cells. It can stimulate T lymphocytes and macrophages to secrete many inflammatory factors, thereby promoting the synthesis of collagen in fibroblasts [46]. In the early stage of pulmonary fibrosis, a large amount of laminin was deposited in the lung tissue, and the level of laminin is often positively correlated with the patient's condition [47]. The experimental results revealed that YYYQ significantly reduced the mRNA expression of *α*-SMA and improved mRNA expression of E-cadherin when compared with BLM group on day 28, in which BLM+YYYQ-H group had better effect than BLM + Pred group. The mRNA expression of laminin and collagen I were both decreased in BLM+ Pred and BLM+YYYQ groups, but YYYQ groups exhibited better therapeutic effect than that of BLM + Pred group. The data from the present study suggested that YYYQ could inhibit TGF-*β*1 mediated EMT, upregulate mRNA expression of *α*-SMA, and downregulate mRNA expression of E-cadherin in BLM-induced pulmonary fibrotic rats.

In conclusion, our study exhibited the effectiveness of YYYQ in delaying the progression of pulmonary fibrosis. However, there were several limitations encountered in this study. For example, on day 14, YYYQ had no significant effect on the mRNA expression of *α*-SMA, E-cadherin, Collagen I, and CTGF, but the therapeutic effect of each index was better on day 28. The reason may be that YYYQ has better therapeutic effects during the latter stage of pulmonary fibrotic rats. This may be due to the latter stage of pulmonary fibrosis, which is marked by increased lung fibers, and reduction in lung capacity has a closer resemblance to the concept of Feiwei in Chinese medicine. As mentioned earlier, Feiwei is a state in which the main pathogenesis is highlighted by deficiency of qi and yin, as well as presence of blood stasis. Hence, the treatment principles of pulmonary fibrosis are nourishing yin and qi and promoting blood circulation, which are in line with the therapeutic effects of YYYQ. Besides, the results also indicated that YYYQ did not reduce the protein concentration of interleukin 18 significantly. From the perspective of Chinese medicine, the pathological state of toxic-heat resembles the state of inflammation. It is postulated that interleukin 18, a proinflammatory cytokine, is not affected by YYYQ significantly, because this decoction contains few herbs which carry the effect of clearing heat and detoxification. This may be also due to the limited role played by interleukin 18 in the process of pulmonary fibrosis; thus, the effect of YYYQ on interleukin 18 is less significant. Our future perspective is to further study the mechanisms on how YYYQ could inhibit TGF-*β*1/Smad signaling pathway in BLM-induced pulmonary fibrotic rats.

## 5. Conclusion

In summary, the result of our study concluded that YYYQ could alleviate the severity of alveolitis and fibrosis on BLM-induced pulmonary fibrosis in rats, inhibit the mRNA expression of TGF-*β*1 and its receptors, Smad3, *α*-SMA, and CTGF, enhance mRNA expression of Smad7 and E-cadherin, and reduce the mRNA expression of collagen such as laminin, collagen I, and hydroxyproline, but the effect on interleukin 18 was not obvious. In other words, YYYQ has the potential of ameliorating progression of pulmonary fibrosis, and the mechanism may be related to suppressing TGF-*β*1/Smad signal pathway and EMT in BLM-induced pulmonary fibrosis of rats.

## Figures and Tables

**Table 1:** Primer information for real-time polymerase chain reaction.

Gene name	Primer Sequence(5′to3′)	Post-primer(5′to3′)
TGF-*β*1	CATTTGGAGCCTGGACACACA	GCTTGCGACCCACGTAGTAGAC
Smad3	GCTGTCTACCAGTTGACTCGCAT	GGGTGCTGGTCACTGTCTGTCT
Smad7	TGTCCAGACGCTGTACCTTCCT	AGTCTTCTCCTCCCAGTATGCCA
T*β*RI	AATGGCGGGGAGAAGAAG	CCGTGGACAGAGCGAGTT
T*β*RII	GATTATGAGCCCCCATTTGGT	AGTGTTCAGGGAGCCGTCTTCT
*α*-SMA	TGACCCAGATTATGTTTGAG	AGATAGGCACGTTGTGAGTC
E-cadherin	GCCAGAGTTTATCCAGGAGGT	TGAGGATGGTGTAGGCGATG
Collagen I	ATCCTGGGTAAAAAGGCAAAGACTA	AAGCGTGCTGTAGGTGAATCG
Laminin	CTGAGGAAACCCTGACCAAT	GGAGTGTTTTTGCTTCATTTTG
GAPDH	CCTTCCGTGTTCCTACCCC	GCCCAGGATGCCCTTTAGTG

**Table 2:** Histopathologic data of each group on 14d and 28d were presented as mean± SD (n=8). Compared with control group ( _ _^*∗*^*p *<0.05,  _ _^*∗∗*^*p* <0.01), compared with BLM group ( _ _^#^*p *<0.05,  _ _^##^*p *<0.01), compared with BLM + Pred group ( _ _^▲^*p *<0.05,  _ _^▲▲^*p *<0.01), and compared with BLM + YYYQ-L group (△*p *<0.05, △△*p *<0.01).

Group	HE		Masson	
	14d	28d	14d	28d
Control	0.10±0.30	0.00±0.00	0.05±0.22	0.03±0.16
BLM	2.11±0.93_ _^*∗∗*^	1.75±0.97_ _^*∗∗*^	1.87±0.81_ _^*∗∗*^	2.43±0.71_ _^*∗∗*^
BLM+ Pred	1.76±0.80_ _^*∗∗*^	1.34±0.87_ _^*∗∗*^	1.73±0.85_ _^*∗∗*^	1.80±0.73_ _^*∗∗*##^
BLM+YYYQ-L	1.77±0.95_ _^*∗∗*^	1.31±0.82_ _^*∗∗*^	1.62±0.86_ _^*∗∗*^	1.63±0.76_ _^*∗∗*##^
BLM+YYYQ-M	1.56±0.91_ _^*∗∗*##^	1.38±0.82_ _^*∗∗*^	1.51±0.89_ _^*∗∗*^	1.65±0.70_ _^*∗∗*##^
BLM+YYYQ-H	1.45±0.79_ _^*∗∗*##^	1.11±0.84_ _^*∗∗*##^	1.08±0.90_ _^*∗∗*##▲▲△△^	1.39±0.64_ _^*∗∗*##▲^

**Table 3:** Data of ELISA assay of each group on day 14 and 28 were presented as mean± SD. Compared with control group ( _ _^*∗*^*p *<0.05,  _ _^*∗∗*^*p* <0.01), compared with BLM group ( _ _^#^*p *<0.05,  _ _^##^*p *<0.01), compared with BLM + Pred group ( _ _^▲^*p *<0.05,  _ _^▲▲^*p *<0.01), and compared with BLM + YYYQ-L group (△*p *<0.05, △△*p *<0.01).

Group	TGF-*β*1	CTGF	Interleukin-18	Hydroxyproline
14d				
Control	498.52±219.73	75.81±6.25	14.71±8.99	9.17±3.40
BLM	1282.41±527.68_ _^*∗∗*^	197.08±67.21_ _^*∗∗*^	27.29±5.86_ _^*∗∗*^	22.20±6.76_ _^*∗∗*^
BLM+ Pred	910.99±573.65	167.48±87.06_ _^*∗*^	22.58±8.19	16.64±2.10_ _^*∗*^
BLM+YYYQ-L	593.06±194.64_ _^##^	165.50±58.84_ _^*∗*^	26.09±3.62_ _^*∗∗*^	13.63±4.78_ _^##^
BLM+YYYQ-M	622.92±211.11_ _^#^	133.57±60.70	22.47±7.85	13.73±5.46_ _^##^
BLM+YYYQ-H	529.98±360.52_ _^##^	139.80±69.29	20.39±5.78	14.05±4.86_ _^##^
28d				
Control	461.20±185.66	73.6±13.28	14.78±7.93	10.37±3.31
BLM	1246.48±256.99_ _^*∗∗*^	204.17±20.16_ _^*∗∗*^	24.79±7.12_ _^*∗∗*^	26.40±5.48_ _^*∗∗*^
BLM+ Pred	678.90±111.96_ _^##^	139.87±29.17_ _^*∗*#^	19.10±3.52	16.82±4.02_ _^*∗*##^
BLM+YYYQ-L	816.98±301.35_ _^*∗*#^	124.85±44.37_ _^##^	22.85±6.00_ _^*∗*^	17.28±6.75_ _^*∗*##^
BLM+YYYQ-M	548.28±321.04_ _^##^	119.87±43.52_ _^##^	20.21±4.28	15.58±2.07_ _^##^
BLM+YYYQ-H	604.26±341.36_ _^##^	121.69±57.49_ _^##^	20.64±4.05	14.46±3.51_ _^##^

**Table 4:** Data of real-time PCR assay of each group on day 14 and 28 were presented as mean± SD. Compared with control group ( _ _^*∗*^*p *<0.05,  _ _^*∗∗*^*p* <0.01), compared with BLM group ( _ _^#^*p *<0.05,  _ _^##^* p *<0.01), compared with BLM + Pred group ( _ _^▲^*p *<0.05,  _ _^▲▲^*p *<0.01), and compared with BLM + YYYQ-L group (△*p *<0.05, △△*p *<0.01).

Group	*α*-SMA	E-cadherin	Laminin	Collagen I
14d				
Control	0.92±0.14	1.10±0.51	0.89±0.12	0.86±0.18
BLM	2.25±1.27_ _^*∗∗*^	0.38±0.43_ _^*∗*^	2.95±0.51_ _^*∗∗*^	2.65±0.60_ _^*∗*^
BLM+ Pred	1.74±0.58	0.44±0.39_ _^*∗*^	2.02±0.59_ _^*∗∗*##^	2.37±1.28_ _^*∗*^
BLM+YYYQ-L	1.49±0.29	0.71±0.16	1.90±0.57_ _^*∗∗*##^	2.08±1.20
BLM+YYYQ-M	1.31±0.66	0.80±0.40	1.66±0.10^##^	1.78±0.46
BLM+YYYQ-H	1.43±0.17	0.91±0.51	1.47±0.42^##^	1.62±0.64
28d				
Control	0.96±0.09	1.23±0.41	0.83±0.16	0.79±0.15
BLM	2.69±0.59_ _^*∗∗*^	0.33±0.29_ _^*∗∗*^	3.23±0.45_ _^*∗∗*^	3.28±1.15_ _^*∗∗*^
BLM+ Pred	1.97±0.58_ _^*∗∗*#^	0.42±0.21_ _^*∗∗*^	2.21±0.41_ _^*∗∗*##^	2.30±0.62_ _^*∗∗*#^
BLM+YYYQ-L	1.59±0.71_ _^##^	0.59±0.36_ _^*∗*^	1.61±0.25_ _^*∗∗*##▲^	2.02±0.60_ _^*∗*#^
BLM+YYYQ-M	1.39±0.34_ _^##^	0.81±0.35	1.50±0.47_ _^*∗*##▲^	1.55±0.60_ _^##^
BLM+YYYQ-H	1.32±0.12_ _^##^	1.00±0.52_ _^#▲^	1.49±0.38_ _^*∗*##▲^	1.40±0.12_ _^##^

**Table 5:** Data of real-time PCR assay of each group on day 14 and 28 were presented as mean± SD. Compared with control group ( _ _^*∗*^*p *<0.05,  _ _^*∗∗*^*p* <0.01), compared with BLM group ( _ _^#^*p *<0.05,  _ _^##^*p *<0.01), compared with BLM + Pred group ( _ _^▲^*p *<0.05,  _ _^▲▲^*p *<0.01), and compared with BLM + YYYQ-L group (△*p *<0.05, △△*p *<0.01).

Group	TGF-*β*1	T*β*RI	T*β*RII	Smad3	Smad7
14d					
Control	1.08±0.16	1.00±0.17	1.06±0.08	0.61±0.27	1.21±0.31
BLM	2.39±0.77_ _^*∗∗*^	3.02±0.79_ _^*∗∗*^	3.10±0.63_ _^*∗∗*^	2.31±0.76_ _^*∗∗*^	0.63±0.13_ _^*∗∗*^
BLM+ Pred	1.85±0.35_ _^*∗*^	2.54±0.52_ _^*∗∗*^	2.37±0.85_ _^*∗∗*^	2.07±0.57_ _^*∗*^	0.94±0.23
BLM+YYYQ-L	1.82±0.55_ _^*∗*^	2.41±0.99_ _^*∗*^	2.15±0.82_ _^*∗*#^	1.54±0.33_ _^*∗*#^	0.94±0.22
BLM+YYYQ-M	1.53±0.29_ _^#^	2.06±0.68_ _^*∗*^	1.32±0.50_ _^##▲^	1.23±0.60_ _^##▲^	1.10±0.39_ _^#^
BLM+YYYQ-H	1.30±0.58_ _^##^	2.10±0.77_ _^*∗*^	2.0±0.87_ _^*∗*#^	1.22±0.20_ _^##▲^	1.00±0.26
28d					
Control	1.10±0.23	1.07±0.60	0.94±0.05	0.88±0.40	1.24±0.40
BLM	2.91±0.58_ _^*∗∗*^	4.12±0.84_ _^*∗∗*^	3.34±0.42_ _^*∗∗*^	2.83±0.43_ _^*∗∗*^	0.50±0.07_ _^*∗∗*^
BLM+ Pred	1.69±0.10^##^	2.80±0.67_ _^*∗∗*##^	1.84±0.24_ _^*∗*##^	2.18±0.32_ _^*∗∗*^	0.97±0.27_ _^#^
BLM+YYYQ-L	1.62±0.59_ _^##^	2.54±0.46_ _^*∗∗*##^	2.14±0.54_ _^*∗∗*##^	1.54±0.52_ _^##^	1.04±0.44_ _^#^
BLM+YYYQ-M	1.40±0.24_ _^##^	1.94±0.60_ _^##^	1.57±0.85_ _^##^	1.25±0.31_ _^##▲^	1.23±0.30_ _^##^
BLM+YYYQ-H	1.19±0.43_ _^##^	1.60±0.46_ _^##▲△^	1.34±0.88_ _^##△^	1.51±0.62_ _^##^	1.08±0.20_ _^#^

## Data Availability

All the data related to this article were available from the corresponding author upon reasonable request.

## Abbreviations

YYYQ: Yangyin Yiqi Mixture
IPF: Idiopathic pulmonary fibrosis
EMT: Epithelial-mesenchymal transition
EMC: Extracellular matrix
BLM: Bleomycin
Pred: Prednisone
TGF-*β*1: Transforming growth factor-*β*1
T*β*RI: Transforming growth factor-*β*1 receptor I
T*β*RII: Transforming growth factor-*β*1 receptor II
CTGF: Connective tissue growth factor
*α*-SMA: *α*-Smooth muscle actin
SD: Standard deviation
H&E: Hematoxylin and eosin.
